# Westminster Hospital and Clapham Common

**Published:** 1914-04-11

**Authors:** 


### The Hospital

The Workers' Newspaper of Administrative Medicine and Institutional Life, Administration, National Insurance and Health.

No. 1450, Vol. LVI. SATURDAY, APRIL 11, 1914. PRICE ONE PENNY.

### WESTMINSTER HOSPITAL AND CLAPHAM COMMON.

On Saturday last the statement was made in the daily press that the governors of the Westminster Hospital have definitely decided to remove to a site at Clapham Common, and that they have instructed a firm of estate agents to dispose of the present site in Broad Sanctuary. This announcement, as it appeared in the papers, was so positively worded that it must be assumed to be, if not definitely official, at least inspired by someone thoroughly versed in the present intentions of the governors. As it stands, it almost has the air of breaking off the negotiations for amalgamation with St. George's, which caused so much interest in London hospital circles last year; but we have reason to believe such an impression would be erroneous. For example, we know—there is no secret about it—that the question of a site divided the two hospitals rather acutely some months ago. But we cannot think that a plan so bold, so advantageous to both hospitals, and so full of promise of increased usefulness to the sick poor of South London can really be wrecked over a small point like this.

The position of the two hospitals can be succinctly stated. Both are placed on exceedingly fine sites in the West of London; both sites have become, by the growth of London, very valuable. Both hospitals have found themselves, as years elapsed, more and more surrounded by houses of a kind such as hospital patients do not live in, and, correspondingly, the classes who do make up the *clientèle* at both hospitals have further and further to come from their homes to obtain treatment. Both hospitals have perceived that their future work can best be carried out by removing into poorer and more densely populated neighbourhoods. Both are in the advantageous position of having sites to sell which will fetch large sums of money towards meeting the expenses of such moves. Further than that, the two hospitals have ancient associations, for St. George's was founded in 1733 by a secession of staff and subscribers from the Westminster, then fourteen years of age. The reunion of the two after so long a severance naturally appealed to the imaginations of the public and the institutional world. More than this, it would be of practical value in the apportionment of hospital beds to the new needs of London, to which the Surrey side of the river now contributes so much.

In one respect a lucky combination of chances seemed to favour the project—namely, in respect of the medical staff appointments. The number of the visiting staff in the suggested joint hospital would inevitably be smaller than in the two hospitals as they at present stand; but, fortunately, the governors were able to forecast certain inevitable retirements and vacancies, which would enable practically all the remaining members of both staffs to be given the same standing as they already had. No personal considerations could be allowed to stand in the way of such a great project as this amalgamation; but it was satisfactory to know that none would arise.

Then with regard to funds, the prospects were not at all unfavourable. St. George's is much the wealthier in invested capital, and the Hyde Park Corner site is more valuable than that at Broad Sanctuary. Excusably, St. George's regarded itself as the senior partner in the enterprise, and wanted a deciding voice in the arrangements. A site at Battersea appealed most to the St. George's governors, one at Clapham to those of the Westminster Hospital. Then came the failure of the efforts to sell the site of St. George's to Mr. Mallaby Deeley, and the subsequent controversies with regard to the management of that hospital. It is no more than bare truth to say that these incidents created a feeling of lessened confidence in the managers. However, the incidents are now closed, and may well be relegated to ancient history. Whether they had much effect on the negotiations for amalgamation, and, if so, what that effect was, we have no idea; but it is hardly possible that there can have been no effect at all. It will be a thousand pities if these domestic affairs should result in the falling through of a fine scheme, and we hope that the danger of this is less than would be suspected from the wording of the paragraph in last Saturday's papers. An authoritative pronouncement on this point from the joint committee which has the matter in hand would be useful at this juncture, if such a thing is practicable without prejudice to the negotiations.

One word more. Rightly or wrongly, it was understood more than a year ago that the Westminster Hospital site was already sold. It is evident from the recent announcement that the site is still for sale; so the inference is either that the rumour referred to was incorrect or else that the sale—like that of St. George's—fell through before its completion. At all events, it would, in our judgment, be most unwise for the Westminster Hospital to be committed to any scheme of rebuilding at Clapham until there is a certainty of selling the present site and of sufficient financial support to carry through the move. And it would also be very poor tactics to repeat the proposal of a year ago to pull down some Jacobean houses on the Clapham site, for it is sure to alienate sympathy and, more important still, subscriptions to the building fund.

